# Case report: Chronic *Candida albicans* meningitis: a rare entity diagnosed by metagenomic next-generation sequencing

**DOI:** 10.3389/fcimb.2024.1322847

**Published:** 2024-04-19

**Authors:** Andrea B. Kuenzli, Mandy D. Müller, Werner J. Z`Graggen, Laura N. Walti, Yonas Martin, Vladimir Lazarevic, Jacques Schrenzel, Alexander Oberli

**Affiliations:** ^1^Department of Infectious Diseases, Inselspital, Bern University Hospital, University of Bern, Bern, Switzerland; ^2^Service d’Infectiologie, Department of Internal Medicine, Neuchâtel Hospital Network, Neuchâtel, Switzerland; ^3^Department of Neurosurgery, Inselspital, Bern University Hospital, University of Bern, Bern, Switzerland; ^4^Department of General Internal Medicine, Inselspital, Bern University Hospital, University of Bern, Bern, Switzerland; ^5^Genomic Research Laboratory, Geneva University Hospitals (HUG) and University of Geneva, Geneva, Switzerland; ^6^Institute for Infectious Diseases, University of Bern, Bern, Switzerland

**Keywords:** *Candida albicans*, chronic meningitis, ventriculitis, metagenomic next-generation sequencing, (1,3)-beta-D-glucan, cerebrospinal fluid

## Abstract

The aetiology of chronic aseptic meningitis is difficult to establish. *Candida* meningitis in particular is often diagnosed late, as cerebrospinal fluid (CSF) work-up and imaging findings are nonspecific. A 35-year-old patient with chronic aseptic meningitis, for which repeated microbiological testing of CSF was unrevealing, was finally diagnosed with *Candida albicans (C. albicans)* meningitis with cauda equina involvement using metagenomic next-generation sequencing (mNGS). This report highlights the diagnostic challenges and the difficulties of treating shunt-associated fungal meningitis.

## Introduction

Chronic meningitis, defined as an inflammation of the meninges lasting for more than four weeks, is an infrequent entity. Its etiology is very diverse, including (opportunistic) infections, neoplastic, autoimmune, and inflammatory as well as drug-induced diseases, and is therefore often difficult to determine, with at least one third of patients remaining undiagnosed. Underlying causes also depend on the population and the geographical area studied, so relative proportions of these etiologies cannot be generalized. For example, in one retrospective study of 79 patients with chronic meningitis, only 44.3% received a definitive diagnosis, 31.6% were bacterial (mostly *Mycobacterium tuberculosis* and *Brucella* spp.) and 10.1% were neoplastic (Bineshfar et al., 2022). Even with invasive diagnostic methods, i.e. brain biopsy, definitive diagnosis is not always achievable; one study of 37 patients with chronic meningitis identified a definitive diagnosis by brain biopsy in 39% of them, the most frequent being sarcoidosis and metastatic adenocarcinoma; the yield rose to 80% when taking into account biopsies obtained from enhancing lesions on magnetic resonance imaging (MRI) only (Cheng et al., 1994).

Chronic *Candida albicans (C. albicans)* meningitis is very rare, and diagnosis typically delayed (Farrugia et al., 2016; Gheshlaghi and Helweg-Larsen, 2020). CSF chemistry and cell counts as well as imaging patterns are nonspecific. Moreover, CSF and blood cultures have a low sensitivity for detecting *Candida* (McGinnis, 1983, Chaussade et al., 2021*).* Alternative diagnoses of chronic progressive meningoencephalitis, including tuberculosis and lymphoma, have similar diagnostic challenges. Therefore, many patients belatedly diagnosed with chronic *Candida* meningitis receive unnecessary, potentially harmful treatments (Voice et al., 1994). Our patient’s extensive microbiological workup was inconclusive and he was initially diagnosed with and treated for presumptive cerebral lymphoma. The correct diagnosis could be established only once cauda equina biopsy grew *C. albicans* on day 78, confirming an earlier result obtained through mNGS.

## Case description

A 35-year-old illicit drug user with a well-controlled HIV infection (CD4+ count 1389 cells/µL) was admitted with a three-months’ history of headaches, fever, and a psychotic state, as well as new-onset diffuse myoclonus and fluctuations in vigilance.

Six months prior to the current admission, a pulmonary small-vessel vasculitis potentially associated with hepatitis C reinfection required treatment with corticosteroids and a course of glecaprevir/pibrentasvir. During that previous hospitalisation, fluconazole was administered for two weeks for a *C. albicans* fungemia following cocaine injection through his central intravenous line, with negative follow-up cultures. Three months later, a psychotic state with risk of self-harm had required a hospitalisation in a psychiatric clinic.

**Figure 1:**
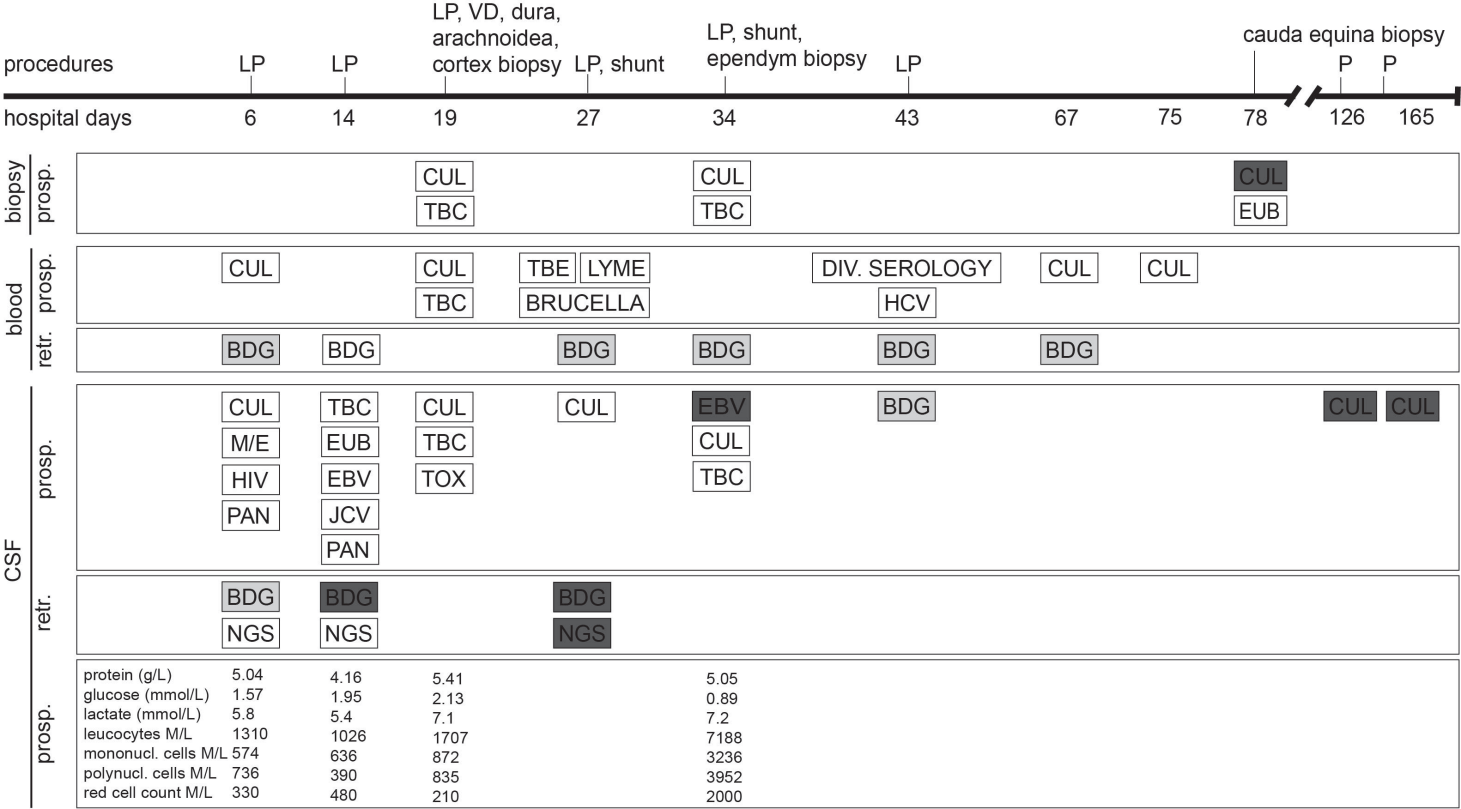
Timeline in hospital days after admission of clinical characteristics, prospective (prosp.) and retrospective (retr.) diagnostic testing. White bars represent negative diagnostic results, light grey bars represent borderline values and dark grey bars represent positive results. (1,3)-β-D-glucan (BDG) cutoff values for CSF were chosen as set for BDG testing in serum. Each BDG test was performed in parallel with both Fungitell and Wako FUJIFILM approach. BDG, (1,3)-β-D-glucan; CUL, culture; EBV, Epstein-Barr virus PCR; EUB, bacterial PCR; HCV, hepatitis C virus PCR; HIV, human immunodeficiency virus PCR; JCV, JC virus (human polyomavirus 2) PCR; LP, lumbar puncture; M/E, BIOFIRE FILMARRAY Meningitis/Encephalitis (ME) panel; NGS, next-generation sequencing; P, CSF puncture; PAN, pan-fungal PCR; TBC, tuberculosis culture and PCR; TBE, tick-borne encephalitis; TOX, toxoplasmosis PCR; VD, ventricular drainage. For details on medical treatment, refer to the **Supplementary File**.

On the current admission, the patient reported headaches, photophobia and intermittent febrile episodes. His treatment consisted of dolutegravir/abacavir/lamivudine, tapered systemic corticosteroids with co-trimoxazole prophylaxis, and opioid substitution. On clinical examination, the patient was found to have intermittent generalised myocloni, fluctuations in vigilance, and meningeal signs. The brain MRI revealed extensive leptomeningeal and ventricular ependymal enhancement, signs of polyradiculitis of cranial nerves V, VII, VIII, and hyperintensity of both caudate nuclei. Analysis of CSF revealed mixed pleocytosis, low glucose, elevated protein and lactate levels, but extensive microbiological testing was unrevealing (**Figure 1**). Empiric treatment for meningoencephalitis consisted of ceftriaxone, amoxicillin, and acyclovir as well as anti-tuberculous therapy including high-dose corticotherapy. Despite this broad treatment, the patient’s neurological state deteriorated.

## Diagnostic assessment

Open biopsies of the meninges and cortex on hospital day 19 showed discrete inflammation, no signs of vasculitis or malignancy, and no bacterial or fungal growth. On day 27, a generalized seizure due to progressive hydrocephalus prompted the insertion of a ventriculo-peritoneal shunt. Progressive widening of the undrained ventricles - suggesting insufficient communication between the ventricles with trapping and compartmentalization of the supra- and infratentorial ventricular system - required additional ventriculo-peritoneal shunts to be inserted in the contralateral, fourth, and both temporal horns of the lateral ventricles. A repeat brain biopsy on day 34 showed a lympho-plasmacellular infiltrate with clonality. Epstein-Barr-Virus (EBV) was detected in CSF, a finding which is often associated with cerebral lymphoma. After extensive discussion in our interdisciplinary lymphoma board, B-cell lymphoma was considered the most likely diagnosis, and chemotherapy with rituximab, methotrexate, cytarabine und thiotepa was administered for suspected cerebral lympho-plasmacellular B-cell lymphoma. mNGS of a CSF sample collected on day 27 identified *C. albicans* (see **Table 1**). This result was dismissed, as repeat microbiological cultures had not revealed any growth, and an alternative diagnosis had been established.

**Table 1:** Number of read pairs obtained by the mNGS analysis and their taxonomic assignments.

BioSample ID (ENA)		SAMEA7388130 LCR_98	SAMEA7388131 LCR_99	SAMEA7388129 LCR_88	SAMEA7388132 LCR_88_98_99
Hospital date		6	14	27	Negative control
Number of read pairs	Total	944071	979841	2493377	145943
	Quality filtered	582319	314872	1277830	26469
	Human	572562	266901	1254920	6249
	Unclassified	5026	29300	13169	8617
	Microbial	4731	18671	9741	11603
Read pairs % in microbial fraction	*Candida albicans*	0	0	**14.29**	0
	*Micrococcus luteus*	**14.12**	**9.44**	**11.43**	**13.09**
	*Cutibacterium acnes*	**27.52**	**14.10**	**11.36**	**30.42**
	*Moraxella osloensis*	1.73	1.53	6.53	**5.71**
	*Kocuria rhizophila*	**4.08**	**8.25**	0.09	4.67

For each sample, values represented in bold type indicate the three most abundant microbial species. For additional information regarding the mNGS pipeline, refer to the **Supplementary File**.Shading was used to highlight the positive, clinically important result.

Increasing lumbar pain prompted a spinal MRI on day 64 revealing leptomeningeal enhancement from the cervical region to the conus. No CSF could be sampled neither by repeated attempts of lumbar punctures nor from the Rickham reservoirs. Fever and fluctuating vigilance persisted. Cerebral MRI on day 76 showed progressive hydrocephalus and unchanged meningeal/ependymal enhancement.

On day 78, an open biopsy at the L3/4 level demonstrated a gelatinous substance between the spinal nerves instead of CSF which grew *C. albicans*, thus confirming chronic *C. albicans* meningitis and shunt-associated infection.

## Therapeutic interventions and outcome

Chemotherapy was discontinued and systemic treatment with liposomal amphotericin-B and flucytosine was started on day 79. Due to good clinical response, therapy was stepped down to oral fluconazole after two weeks and the patient was sent for rehabilitation.

One month later, the patient was re-admitted because of neurological deterioration due to shunt dysfunction. CSF cultures still grew *C. albicans*. Antifungal therapy was escalated back to liposomal amphotericin-B and flucytosine. Intracranial shunt removal was repetitively discussed but not attempted because of assumed high perioperative bleeding risk due to inflammation.

The patient deteriorated, presenting short-term memory loss and psychiatric issues. CSF culture from day 165 after the first admission - day 85 of antifungal treatment - yet again grew *C. albicans* (with unchanged minimal inhibitory concentrations for antifungals used), and lumbar MRI showed progression. After interdisciplinary discussion and extensive information of the patient’s parents, starting on day 177, liposomal amphotericin-B was administered intrathecally through a spinal catheter with a dosage of 0.5 mg/day, in order to achieve a concentration ten times above the minimal inhibitory concentration (0.25 mg/L) in an estimated CSF-volume of 250 mL. Nevertheless, repeat MRI showed progressive enhancement surrounding the ventriculo-peritoneal shunts, justifying to attempt shunt removal on day 182, despite high perioperative risk: all but one shunt catheter could be extracted without complications. Catheter cultures were negative. Progressive hydrocephalus and clinical deterioration prompted the insertion of two external ventricular drainages on day 194, allowing additional amphotericin administration directly into the ventricles (see **Supplementary Figure 1**).

Despite spinal and intraventricular administration of amphotericin, the patient deteriorated. MRI showed progressive enhancement, and increasing brain oedema. After discussion with the patient’s parents, we opted for palliative care, and the patient died 208 days after his first admission.

## Discussion

Chronic meningitis due to *C. albicans* has been described several months after a candidemia (Porter et al., 1996). Our patient was at risk for candidemia because of his intravenous drug use (Lyons et al., 2015; Poowanawittayakom et al., 2018) and due to prolonged corticosteroid therapy (Goralska et al., 2018). Candidemia had been diagnosed six months prior to the index hospitalization and could have resulted in dissemination to the meninges. The personality changes and fever over a three-month period might therefore be interpreted as early signs of chronic *C. albicans* meningitis (Sarmiento et al., 1994; Porter et al., 1996). This hypothesis is supported by identical internal transcribed spacer sequences in the *C. albicans* strains of the initial blood culture, the biopsy of the cauda, and CSF.

Although mNGS revealed *C*. *albicans* in a culture-negative sample on day 27, two earlier CFS samples, retrospectively analyzed by mNGS and pan-fungal PCR, tested negative. This may be due to a low fungal load in CSF (Verduyn Lunel et al., 2004). Indeed, in the mNGS-positive CSF sample, the number of reads assigned to this organism barely exceeded that of the most common contaminant *Cutibacterium acnes*. The CSF volume (600 µL) used for DNA extraction from undamaged microbial cells was three times that of the first two CSF samples. This is in line with the observation that relatively high CSF volumes (> 5ml) are needed for culture-based detection of fungi (McGinnis, 1983). Diagnosis of chronic fungal meningitis by mNGS has recently been reported in a limited number of cases (Wilson et al., 2018; Cao et al., 2021). A prospective study of patients with meningitis and encephalitis (among whom 13.7% presented an acute exacerbation of a chronic condition) showed improved diagnosis using mNGS. Notably, 13 infections (constituting 22% of the 58 infection cases) were diagnosed only by this method (Wilson et al., 2019). mNGS was falsely negative in 26 cases: in 11 infections identified as negative by conventional microbiological testing and confirmed using serology, in 7 infections diagnosed from samples other than CSF, and in 8 infections exhibiting pathogen titers below the threshold. Another study evaluating diagnostic test accuracy of a mNGS assay in CSF samples of 95 patients with acute meningitis or encephalitis found a sensitivity of 73% and specificity of 99% compared to original clinical test results (Miller et al., 2019).

Retrospective testing revealed slightly elevated serum (1,3)-β-D-glucan (BDG) levels in the first week of admission. In week 2, serum BDG levels were just below the negative cut-off, whereas CSF BDG levels showed levels >500 pg/mL (Fungitell) and 72.4 pg/mL (Wako FUJIFILM). These findings indicate that this *Candida* meningitis is rather a manifestation of a disseminated candidiasis than an iatrogenic shunt infection. CSF BDG levels remained constantly high, also on antifungal therapy. This is in accordance with previous studies in patients with cerebral fungal infections but also in non-neutropenic rabbits, which revealed that serum BDG levels respond promptly to antifungal therapy, whereas the CSF BDG levels remain elevated, reflecting persistent CNS infection (Petraitiene et al., 2008; Salvatore et al., 2016).

The mNGS result of *C. albicans* was initially wrongly dismissed, as repeat liquor cultures did not show any growth, and an alternative presumptive diagnosis – CNS lymphoma – was just then established, based on the histopathology of the brain biopsy and the clonality of CSF lymphocytes. The fact that an underlying HIV infection is clearly associated with a higher incidence of primary CNS lymphoma and the presence of EBV in the CSF of this HIV patient – a finding often identified in the setting of these lymphomas – also contributed to the misdiagnosis of CNS lymphoma.

Treatment of *C. albicans* meningitis is based on expert opinion (Pappas et al., 2016; Schwartz et al., 2018). Removal of indwelling cerebral devices is highly recommended. Intrathecal treatment with amphotericin is reserved for situations where CNS devices cannot be removed, or for patients who have not responded to systemic antifungal therapy. Toxicity (headaches, nausea, and vomiting) is the limiting factor of intrathecal application. However, our patient tolerated the treatment well. In our case, despite removal of four of the five shunts and intrathecal application of antifungals, we were not able to halt disease progression. In retrospect and in view of the extensive disease and high fungal load, the patient should have benefitted of earlier intrathecal antifungal therapy and earlier shunt removal.

Our patient’s chronic *C. albicans* meningitis was fatal because of delay in diagnosis and subsequent shunt infection which made eradication of the fungus impossible. Microbiological work-up did not identify any other pathogen but EBV. This virus can also be found concomitantly with other CNS pathogens, without a clear pathogenic role, mainly in severely immunocompromised patients (Martelius et al., 2011). Also, the role of the presumed CNS lymphoma remains uncertain: although histopathology showed a lympho-plasmacellular infiltrate with clonality, there was no clinical response to chemotherapy but only progression of the meningeal changes.

Unfortunately, given the patient’s neurocognitive state and his passing, we are not able to give any patient’s perspective on this case.

## Conclusion

In conclusion, diagnosis and treatment of chronic *Candida* meningitis remain challenging. Timely diagnosis and presumably also early CNS device removal are of utmost importance in order to avoid unfavorable outcomes. Ideally, large CSF volumes should be cultivated in order to avoid false negative results. If standard microbiological work-up of CSF cannot identify a pathogen, BDG testing and mNGS of CSF as well as taking a CNS biopsy, if feasible, should be considered.

## Data availability statement

mNGS data have been deposited within the European Nucleotide Archive (ENA), under the study accession number PRJEB39758, link: https://www.ebi.ac.uk/ena/browser/text-search?query=PRJEB39758.

## Ethics statement

The case report was conducted in accordance with the local legislation and institutional requirements. The participants' next of kin provided their written informed consent to participate in this case report. Written informed consent was obtained from the participants' next of kin for the publication of any potentially identifiable images or data included in this article.

## Author contributions

AK: Writing – original draft, Writing – review & editing. MM: Writing – original draft, Writing – review & editing. WZ: Writing – review & editing. LW: Writing – review & editing. YM: Writing – review & editing. VL: Writing – review & editing. JS: Writing – review & editing. AO: Writing – original draft, Writing – review & editing.
