# Supplementary figures and images for: Case report: Chronic Candida albicans meningitis: a rare entity diagnosed by metagenomic next-generation sequencing
Source: Front Cell Infect Microbiol. 2024 Apr 19;14:1322847. doi: 10.3389/fcimb.2024.1322847 (PMC11066203; doi:10.3389/fcimb.2024.1322847)

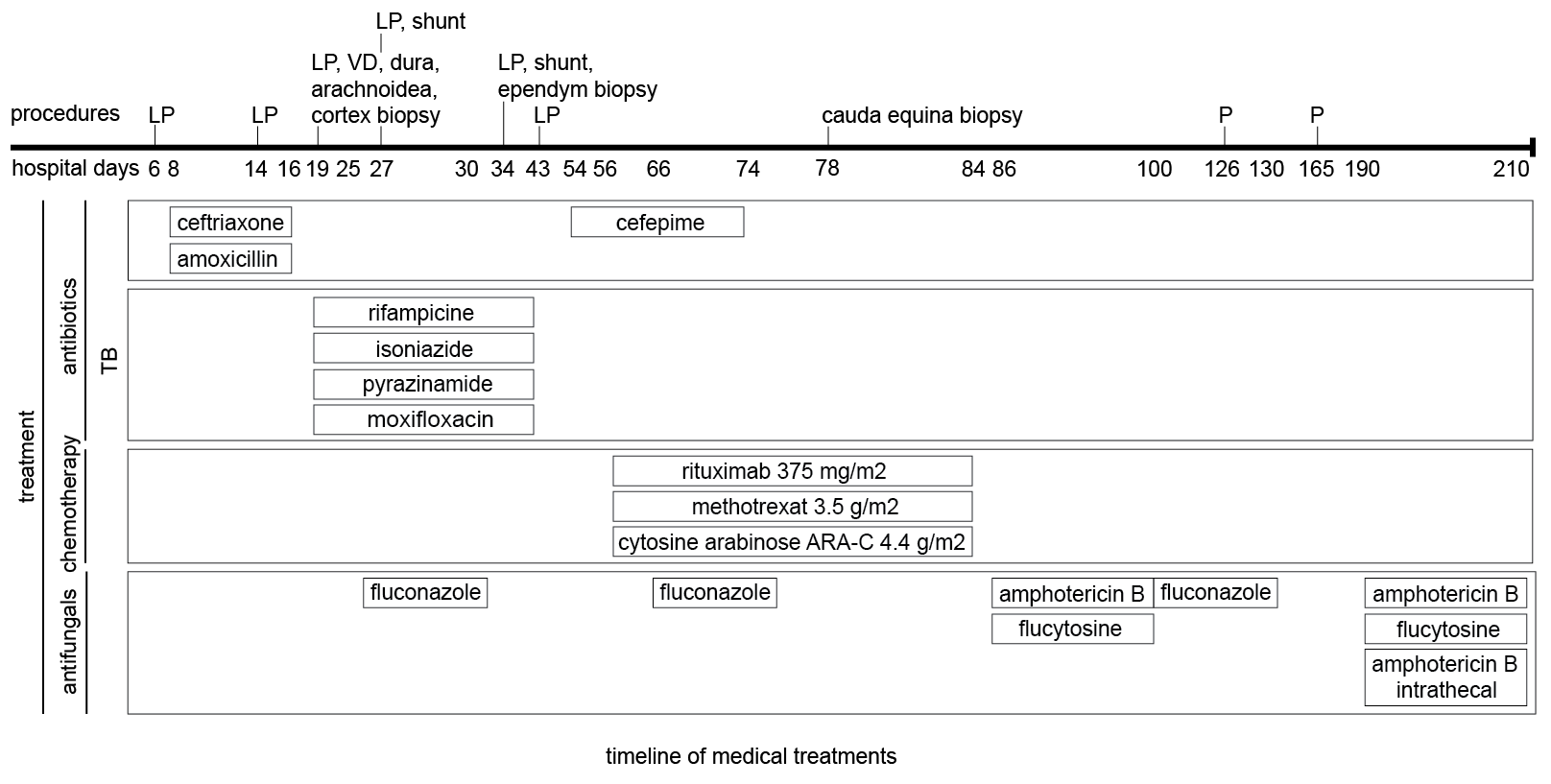

Supplement: Supplementary file 1 [file Image_1.tiff]
