# Supplementary material for: Case report: Chronic Candida albicans meningitis: a rare entity diagnosed by metagenomic next-generation sequencing
Source: Front Cell Infect Microbiol. 2024 Apr 19;14:1322847. doi: 10.3389/fcimb.2024.1322847 (PMC11066203; doi:10.3389/fcimb.2024.1322847)
Supplement: Supplementary file 2 [file Presentation_1.pdf]

Supplement

mNGS pipeline

DNA extraction. DNA extraction was carried out on 200 µL cerebrospinal fluid (CSF) samples for the first two and 600 µL for the third time point samplings. Prior to the extraction process, the samples were filtered through a 100-µm cell strainer (Corning, Corning, NY, USA), which was subsequently washed twice with 200 µL SU buffer from the Ultra-Deep Microbiome Prep kit (Molzym, Bremen, Germany). The DNA extraction protocol followed the manufacturer's instructions for liquid samples using the Ultra-Deep Microbiome Prep kit. A negative extraction control was performed by substituting the clinical sample with the SU buffer.

DNA sequencing. Metagenomic libraries were prepared from 30 µL of DNA extracts using Nextera DNA Flex Library Prep (Illumina, San Diego, USA) with 12 amplification cycles. The libraries were sequenced (2×151) on an Illumina iSeq 100 System instrument.

Bioinformatics analysis. We used Trimmomatic v.0.36 package [1] for the following: (i) the removal of bases corresponding to standard Illumina adapters; (ii) trimming bases from the start or end of a read, if below a quality threshold of 5; and (iii) the trimming of low-quality ends within any 20-base sliding window with an average Phred quality <30. Reads with a length <90 bases after the trimming step were excluded. Additionally, potential artificial replicate reads were filtered out using a custom script, available at https://github.com/GRL-HUG/duplicates.

Reads matching the human genomic sequence were identified using CLARK [2] v.1.2.5 with default parameters and the GRCh38.p7 database [3,4]. After removal of human-associated reads, the data were deposited to European Nucleotide Archive (ENA) database (study number PRJEB39758). The remaining reads were classified at the species level using CLARK (with parameters -m 0 -c 0.8) against the database of representative/reference microbial (bacterial, archaeal, fungal and viral) genomes [4]

1. Bolger AM, Lohse M, Usadel B (2014) Trimmomatic: a flexible trimmer for Illumina sequence data. Bioinformatics 30: 2114-2120.
2. Ounit R, Wanamaker S, Close TJ, Lonardi S (2015) CLARK: fast and accurate classification of metagenomic and genomic sequences using discriminative k-mers. BMC Genomics 16: 236.
3. International Human Genome Sequencing Consortium (2001) Initial sequencing and analysis of the human genome. Nature 409: 860.
4. Pruitt KD, Tatusova T, Maglott DR (2007) NCBI reference sequences (RefSeq): a curated non-redundant sequence database of genomes, transcripts and proteins. Nucleic Acid Research 35: D61-D65.
